# Relationship between sensation seeking, alcohol problems and bulimic symptoms: a community-based, longitudinal study

**DOI:** 10.1007/s40519-021-01193-6

**Published:** 2021-04-26

**Authors:** Leon Hirvelä, Pyry N. Sipilä, Anna Keski-Rahkonen

**Affiliations:** Department of Public Health, University of Helsinki, Helsinki, Finland

**Keywords:** Sensation seeking, Bulimic symptoms, Alcohol problems, Community-based, Longitudinal

## Abstract

**Purpose:**

The association of bulimic symptoms with sensation seeking is uncertain; however, both behaviors have been linked to alcohol problems. We assessed in a longitudinal, community-based setting whether sensation seeking in adolescence is associated with bulimic symptoms in early adulthood, also accounting for alcohol problems.

**Methods:**

Finnish men (*N* = 2000) and women (*N* = 2467) born between 1974–1979 completed Zuckerman’s sensation seeking scale (SSS) at age 18. Alcohol problems (Malmö-modified Michigan alcoholism screening test (Mm-MAST) and bulimic symptoms [eating disorder inventory-2, bulimia subscale (EDI-Bulimia), population and clinical scoring systems] were defined at age 22–27. We examined relationships between SSS, Mm-MAST, and EDI-Bulimia using Pearson’s correlation coefficient (*r*) and linear regression.

**Results:**

Alcohol problems were moderately correlated with sensation seeking and bulimic symptoms (population scoring) among women and men (*r* = 0.21–0.31). The correlation between sensation seeking and bulimic symptoms (population scoring) was weak among men (*r* = 0.06, *p* = 0.006) and even weaker and non-significant among women (*r* = 0.03, *p* = 0.214). Adjustment for alcohol problems removed the association between sensation seeking and bulimic symptoms among men. Furthermore, there were no significant correlations between sensation seeking and bulimic symptoms when assessing EDI-Bulimia clinical scoring.

**Conclusion:**

Sensation seeking and bulimic symptoms were not associated among women. The association between sensation seeking and bulimic symptoms among men was entirely attributable to increased alcohol problems among those with higher sensation seeking. While this association may be important on the population level, its clinical significance may be minor.

**Level of evidence:**

Level III, well-designed cohort study.

## Introduction

Personality traits that help to identify individuals with a high risk of future eating-related problems could be useful in clinical practice. Knowledge of these traits could be used to guide prevention programs targeting adolescents at the highest risk of serious eating disorders.

Impulsivity is a broad personality trait associated with bulimia nervosa, binge-eating disorder, and related behaviors [1, 2]. It is associated with an increased frequency of engaging in these behaviors and higher problem levels of the behaviors [2]. Based on the widely accepted five-factor model of personality [3], it has been proposed that impulsivity consists of four distinct personality traits: urgency, lack of planning (or premeditation), lack of perseverance (or persistence) and sensation seeking [4]. Sensation seeking is further defined as “a need for varied, novel, and complex sensations and experiences and the willingness to take physical and social risks for the sake of such experiences” [5]. It is a relatively heritable trait with reported heritability estimates of 0.61 for females and 0.65 for males from a twin study [6]. Sensation seeking is more characteristic of bingeing and or purging type behaviors (bulimia nervosa and binge-eating disorder) than of restricting-type behavior (anorexia nervosa) [7]. Sensation seeking is also associated with alcohol problems [8–10].

We are aware of only a few community-based studies that have assessed the relationship between sensation seeking and bulimic symptoms. Some of them have examined only women [11], others have examined the relationship only cross-sectionally [10]. Few studies have been truly longitudinal and community-based, also taking men into account [12, 13]. Among them, some have been limited to a short follow-up time in early puberty [13] and others have examined a period from childhood to adolescence without considering early adulthood [12]. Most of the existing community studies have shown either no association between sensation seeking and bulimic symptoms [10, 11, 13] or a mild negative correlation [12], and only one with a cross-sectional setting a positive correlation even after controlling for other psychiatric comorbidities such as victimization experiences and post-traumatic stress disorder (PTSD) [14]. These findings differ from some studies based on clinical samples [1, 15] or other limited populations, such as university students [2, 16–18], which indicate that sensation seeking is positively correlated with bulimic symptoms. However, when comparing these results to the present study, it is notable that only one of the previous studies used the eating disorder inventory-2 (EDI-2) Bulimia subscale to measure bulimic symptoms [16]. In addition, the somewhat conflicting findings may not only be due to the diverse study samples and bulimic symptomatology instruments but because of various instruments assessing sensation seeking as well. Also, some sensation-seeking subscales, such as the disinhibition subscale, might be better in predicting bulimic or binge-type symptoms [14] than the full scale. To sum up, we do not know whether sensation seeking in adolescence is associated with later bulimic symptoms in adulthood.

The role of sensation seeking in the development of future alcohol problems is much better understood. Several previous community-based, longitudinal studies state that sensation seeking is an important factor predicting future alcohol use [8, 9]. However, these studies have not examined comorbid bulimic symptoms. Guller et al. [13] considered alcohol problems when examining the association between sensation seeking and bulimic symptoms in their longitudinal study, but they found no prospective correlation probably due to their study participants’ young age. Two recent cross-sectional studies conducted in student or mostly student populations identified a positive association between sensation seeking, bulimic symptoms, and alcohol problems [17, 18]. As sensation-seeking seems to be an antecedent to alcohol problems [19, 20], alcohol problems could mediate the effects of sensation seeking on bulimic symptoms, but further studies are needed to clarify the longitudinal aspects and relevance in the general population. We know also that individuals with diagnosed bulimia nervosa and other eating disorders report more alcohol problems than do individuals without eating disorders [21]. Alcohol use disorder, bulimia nervosa, and binge-eating disorder are all fairly prevalent psychiatric problems in Finland (lifetime prevalences for women and men 2.5 and 13.2%, 2.4 and 0.16%, and 0.6 and 0.3%, respectively) [22, 23]. Since alcohol use disorder is more prevalent among men, and bulimia nervosa and binge-eating disorder more prevalent among women, it is reasonable to assess the relationship between sensation seeking, bulimic symptoms, and alcohol problems separately for women and men.

Our aim was to assess in a nationwide longitudinal sample of adolescents and young adults whether sensation seeking is associated with later bulimic symptoms. We also examined whether this association is attributable to alcohol problems.

## Methods

### Participants

The population of the present study consists of participants of FinnTwin16, a nationwide longitudinal cohort study assessing health behaviors in twins and their families. FinnTwin16 identified most live twin births in Finland in 1974–1979 from the Central Population Register [24]. Baseline self-report questionnaires were sent to the twins and their parents as the twins turned 16 years old (wave one). Follow-up questionnaires were sent when the twins reached the ages of 17 (wave two), 18.5 (wave three), 22–27 (wave four) and 31–37 years (wave five). Data collection and analysis were approved by the ethics committee of the Department of Public Health of the University of Helsinki and the institutional review board of Indiana University.

Our analyses were based on 5427 responders who participated in both waves three and four. The effective sample comprised 4467 participants (2467 women and 2000 men, 82.3% of the original sample) with complete data on the relevant variables [24].

### Measures

#### Bulimic symptoms

Bulimic symptoms were screened at the age of 22–27 (wave four, mean age 24.4, standard deviation 0.87, range 22.8–27.7) using the EDI-Bulimia subscale of eating disorder inventory-2 (EDI-2) [25]. EDI-Bulimia is a sum of seven items, each with six response options from never to always. There are two different scoring systems for EDI-Bulimia: the “0–3 scoring system” and “1–6 scoring system”. We refer to these as clinical scoring and population scoring, respectively. Clinical scoring is scored from 0 to 3, so that the three most asymptomatic response options are scored as zero and the latter three from 1 to 3. Population scoring is scored from 1 to 6, higher scores indicating more severe bulimic symptomatology. The Clinical scoring is more commonly used and is assumed to make a better differentiation between the ill and healthy [25]. However, as it scores half of the response options as zero, it misses variation on the level of subclinical eating disorder symptomatology. For this reason, we utilized both population scoring and clinical scoring of EDI-Bulimia to one) assess the relationship between sensation seeking and a wide spectrum of bulimic symptoms and two) to evaluate the clinical significance of this relationship. We evaluated bulimic symptoms at symptom level rather than with eating disorder diagnoses because there were not enough men with diagnoses of bulimia nervosa in this sample. Utilizing the continuous EDI-Bulimia subscale, we were able to examine bulimic symptoms in both women and men.

#### Sensation seeking

Our measure of sensation seeking comprised 27 self-report items drawn from all subscales of the sensation seeking scale (SSS) administered at the age of 18.5 (wave three, mean age 18.6, standard deviation 0.17, range 18.3–19.4) [5, 6]. The item format of SSS is a forced choice of two alternatives: one stands for sensation seeking, while the other does not.

#### Alcohol problems

Information on alcohol problems was gathered at the age of 22–27 (wave four) using an originally nine-item self-report, the Malmö modified Michigan alcoholism screening test (Mm-MAST) [26]. The original Mm-MAST was designed for determining current drinking patterns and problems in Nordic countries. The scale used in the present study had two additional items to increase its sensitivity for alcohol use disorder [27].

### Data analysis

Student’s *t*-test was used to evaluate differences in the means of predictor and outcome variables between women and men, while cross-sectional and longitudinal relationships between these variables were assessed using Pearson’s correlation coefficients and their *p* values. Finally, we explored the potential association between sensation seeking and bulimic symptoms (EDI-Bulimia population scoring) with linear regression models, also accounting for alcohol problems and interaction between sensation seeking and gender. All of the aforementioned analyses were adjusted for clustered sampling within the twin pairs using standard methods in the statistical software package Stata 16.0.

## Results

Table 1 shows the distribution of the examined variables by gender. We found no significant difference between the sensation seeking scale scores of men and women (*p* = 0.109). Men had significantly higher scores for alcohol problems (Mm-MAST, *p* < 0.001), while women scored significantly higher for bulimic symptoms both with clinical (*p* < 0.001) and population (*p* < 0.001) scoring systems for EDI-Bulimia.

**Table 1:** Means, medians and *p* values for the difference in means in the predictor and outcome variables between genders

	Mean (SD)		Median		*p*
	Male	Female	Male	Female	
SSS	13.4 (4.7)	13.2 (4.6)	14	13	0.109
Mm-MAST	4.4 (2.9)	3.2 (2.4)	4	3	** < 0.001**
EDI-Bulimia population scoring (1,2,3,4,5,6)	9.8 (2.6)	11.7 (4.4)	9	11	** < 0.001**
EDI-Bulimia clinical scoring (0,0,0,1,2,3)	0.33 (0.95)	0.61 (1.6)	0	0	** < 0.001**

*SD* standard deviation, *SSS* sensation seeking scale, *Mm-MAST* Malmö-modified Michigan alcoholism screening test, *EDI*-*Bulimia* Bulimia subscale of eating disorder inventory-2

Statistically significant *p* values (*p* < 0.05) are highlighted with bold emphasis

Pearson’s correlation coefficients between the variables are shown in Table 2. Sensation seeking was moderately associated with later alcohol problems both among men (*r* = 0.31, *p* < 0.001) and women (*r* = 0.27, *p* < 0.001). Alcohol problems were moderately associated with bulimic symptoms (*r* = 0.22 for men and 0.21 for women). Sensation seeking was weakly associated with later bulimic symptoms among men when EDI-Bulimia population scoring was assessed (*r* = 0.06, *p* = 0.006), but the association turned non-significant when assessing the clinical scoring (*r* = 0.03, *p* = 0.166). The respective association among women was non-significant regardless of the scoring system (*r* = 0.02–0.03, *p* = 0.214–0.286).

**Table 2:** Pearson’s correlation coefficients and the respective *p* values between the variables of interest

	SSS	Mm-MAST	EDI-Bulimia population scoring (1,2,3,4,5,6)	EDI-Bulimia clinical scoring (0,0,0,1,2,3)
SSS		**0.31** ***p * < 0.001**	**0.06** ***p * = 0.006**	0.03 *p* = 0.166
Mm-MAST	**0.27** ***p * < 0.001**		**0.22** ***p*** **< 0.001**	**0.11** ***p* = 0.014**
EDI-Bulimia population scoring (1,2,3,4,5,6)	0.03 *p* = 0.214	**0.21** ***p < 0.001***		**0.70** ***p* < 0.001**
EDI-Bulimia clinical scoring (0,0,0,1,2,3)	0.02 *p* = 0.286	**0.15** ***p* < 0.001**	**0.80** ***p*** **< 0.001**	

Above the diagonal line: men, Below the diagonal line: women

*SSS* sensation seeking scale, *Mm-MAST* Malmö modified Michigan alcoholism screening test, *EDI-Bulimia* Bulimia subscale of eating disorder inventory-2

The statistically significant results (*p* < 0.05) are highlighted with bold emphasis

Results of the (multiple) linear regressions are shown in Table 3. In a model that pooled both men and women, there was no significant association between sensation seeking at age 18 and bulimic symptoms (EDI-Bulimia population scoring) at age 22–27. However, when adjusted for gender, sensation seeking was significantly associated with bulimic symptoms (*β* = 0.029, *p* = 0.018). Additionally, the female gender was strongly associated with increased bulimic symptoms.

**Table 3:** Linear regression models detailing the association between sensation seeking and EDI-Bulimia and accounting for alcohol problems

			Model summary						Regression coefficients			
	Type of model	Dependent variable	Gender	Sample size	Degrees of freedom	R^2	*p*	Independent variable	*β*	*t*	95% CI	*p*
Model pooling men and women	Basic model	EDI-Bulimia (pop. scoring)	Both	4503	F (1, 2571) = 3.71	0.0009	0.054	SSS	0.025	1.93	−0.001–0.050	0.054
	Adjusted model	**EDI-Bulimia** **(pop. scoring)**	Both	4503	F (2, 2571) = 144.23	0.063	** < 0.001**	**SSS**	**0.029**	**2.36**	**0.005–0.05**	**0.018**
								**Female gender**	**1.89**	**16.94**	**1.67–2.11**	** < 0.001**
Model with women only	Basic model	EDI-Bulimia (pop. scoring)	Women	2490	F (1, 1743) = 1.54	0.001	0.214	SSS	0.025	1.24	−0.015–0.065	0.214
Model with men only	Basic model	EDI-Bulimia (pop. scoring)	Men	2013	F (1, 1453) = 7.66	0.004	**0.006**	**SSS**	**0.034**	**2.77**	**0.010–0.058**	**0.006**
	Adjusted model	**EDI-Bulimia** **(pop. scoring)**	Men	2000	F (2, 1447) = 55.26	0.056	** < 0.001**	SSS	−0.008	−0.59	−0.033–0.018	0.552
								**Mm-MAST**	**0.209**	**9.96**	**0.168–0.250**	** < 0.001**

The significant results are highlighted with bold emphasis

*EDI-Bulimia* (pop. scoring) Bulimia subscale of eating disorder inventory-2 (population scoring (1,2,3,4,5,6)), *SSS* sensation seeking scale, *Mm-MAST* Malmö modified Michigan alcoholism screening test, *CI* confidence interval, corrected for clustered sampling

We then conducted the regression models separately for women and men. There were no significant findings between sensation seeking and bulimic symptoms among women. Among men, sensation seeking was significantly associated with bulimic symptoms (*β* = 0.034, *p* = 0.006). However, the regression coefficients for the association of sensation seeking with bulimic symptoms did not differ statistically significantly between women and men (*p* for interaction 0.70). Furthermore, this association among men faded when alcohol problems were taken into account (adjusted *β* = −0.008, *p* = 0.552). Respectively, alcohol problems were significantly associated with bulimic symptoms among men even when the model was adjusted for sensation seeking (*β* = 0.209, *p* < 0.001).

## Discussion

In this longitudinal, community-based study that spanned from adolescence to young adulthood, we found that sensation seeking at the age of 18 was weakly associated with bulimic symptoms at age 22–27 among men. This association was attributable to problematic alcohol use. In contrast, we detected no association between sensation seeking and bulimic symptoms among women. However, when bulimic symptoms were measured with a stricter clinical scoring of EDI-Bulimia, the associations between sensation seeking and bulimic symptoms were statistically non-significant even in men. Moreover, both men and women who had more drinking problems reported more bulimic symptoms, and sensation seeking was related to heavier alcohol use among both men and women.

Our results regarding the association between sensation seeking and later alcohol problems were in line with previous studies [8, 9]. Surprisingly, our results about the role of sensation seeking in later bulimic symptoms were somewhat different from previous community-based, longitudinal studies [12, 13]. Those stated that sensation seeking has either no association with later bulimic symptoms [13] or that it is a weakly protective factor [12]. However, our findings are supported by cross-sectional meta-analyses based on clinical samples [1, 15] and other studies with limited populations, such as university students [2, 16–18], which all show that sensation seeking is positively correlated with bulimic symptoms. Previous studies have not considered the possible gender differences. Our results bring novel information about the role of sensation seeking as a risk factor for subclinical bulimic symptoms among men.

We followed our population from adolescence to young adulthood. The time frame of our study differs from previous longitudinal, community-based studies that have examined the association between sensation seeking and later bulimic behavior [12, 13]. They looked into a short period of time in early puberty [13] or followed the development from childhood to adolescence without considering adulthood [12]. Furthermore, they used other instruments to measure bulimic symptomatology than the present study. It is possible that in large community-based samples the association between sensation seeking and later bulimic symptoms are detected only with measurements sensitive for subclinical bulimic symptoms, such as the population scoring of EDI-Bulimia. Together with previous studies, our study indicates that sensation seeking may start playing a contributory role in the development of bulimic symptoms of young adult men from late adolescence onwards, not earlier on and that women may not be affected by this same risk factor.

Clinicians should keep in mind that alcohol use disorder and bulimia nervosa often coexist with other psychiatric conditions, such as PTSD and major depression [28]. Evidence for the coexistence of alcohol problems and bulimic symptoms were found in the present study, as these were moderately correlated with each other. In addition to this co-occurrence of disorders, sensation seeking is likely to be an antecedent to at least alcohol problems [19, 20].

Our study had some limitations. First, sensation seeking, alcohol problems, and bulimic symptoms were not consistently evaluated at all waves of follow-up. This makes it impossible to resolve the temporal order of the onset of these traits. We do not know whether sensation seeking increased bulimic symptoms, or whether sensation seeking, alcohol problems, and bulimic symptoms were stable across time. The same uncertainty affects our adjusted models. We do not know whether alcohol problems mediated the association between sensation seeking and bulimic symptoms or whether bulimic symptoms preceded the onset of alcohol problems.

Second, although we measured bulimic symptoms among women and men, we were unable to examine the association of sensation seeking with clinical eating disorder outcomes across genders, because clinical eating disorders were too rare among men [29]. The EDI-2 Bulimia subscale is not an ideal instrument to assess the presence of binge-type eating disorders, especially among men [30]. Third, we investigated only one personality trait of the impulsivity scale: sensation seeking. According to a cross-sectional meta-analytic review, sensation seeking, along with lack of planning, is the second strongest component of impulsivity associated with bulimic symptoms, whereas urgency is the strongest. [1]. Future longitudinal studies should replicate and expand our findings by including all impulsivity traits—sensation seeking, urgency, lack of planning, and lack of perseverance—when considering the potential of personality traits to predispose for the development of bulimic symptoms. Nevertheless, we think that it is important to study sensation seeking because it is a partial mediator of genetic risk of alcohol problems [8], and alcohol problems are encountered more severely by individuals with eating disorders, especially bulimia nervosa, than people without eating disorders [21].

Fourth, the design of our study had a significant limitation in not accounting for other comorbidities than alcohol problems that could alter the relationship between sensation seeking and bulimic symptoms. PTSD and its symptoms along with traumatic experiences have been associated with both alcohol problems and binge-type behavior in previous studies [31, 32]. These traits need to be considered in future longitudinal studies assessing the associations between impulsive personality traits, bulimic symptoms, and alcohol problems.

However, we believe that the limitations of our study design are offset by its longitudinal and nationwide scope. Our large sample that also included men, older adolescents, and young adults helped us to reexamine and expand earlier findings. Adequately powered community-based studies are able to provide highly reliable estimates and generalizable findings. In addition, utilizing both population scoring and clinical scoring of EDI-Bulimia helped us to clarify the association of sensation seeking with both subclinical and more severe bulimic symptoms.

In conclusion, we observed a modest association between sensation seeking and population-level bulimic symptoms among men but the respective association was not significant among women. However, the clinical significance of sensation seeking in predicting later bulimic symptoms may be small even in men. Interestingly, the association between sensation seeking and bulimic symptoms among men was wholly accounted for by increased alcohol problems among those with higher sensation seeking.

### What is already known on this subject?

The association between sensation seeking and bulimic symptoms is uncertain: the few longitudinal, community-based studies show either no association or a mild negative correlation between these traits. On the other hand, studies based on limited populations such as clinical samples or university students show a positive correlation between sensation seeking and bulimic symptoms. The role of sensation seeking in later alcohol problems appears to be clear: sensation-seeking predicts heavier alcohol use. Bulimic symptoms and alcohol problems often co-exist, but no longitudinal studies have examined the role of sensation seeking in predicting both of these traits in adolescent or adult populations.

### What this study adds?

This study shows that sensation seeking in late adolescence was weakly correlated with population-level bulimic symptoms in young adult men, but the respective correlation was not significant among women. The association between sensation seeking and bulimic symptoms among men was entirely attributable to increased alcohol problems among those with higher sensation seeking and might not be detectable using the routine clinical scoring of the eating disorder inventory (EDI).

## Data Availability

The authors have no permission to share the data of this study.
